# Mechanical septal debulking to enable left bundle branch pacing through dense septal scar: A bailout technique

**DOI:** 10.1016/j.hrcr.2026.07.003

**Published:** 2026-07-11

**Authors:** Martín Negreira-Caamaño, Agustín Pastor-Fuentes, Joaquin Jesús Alonso-Martín

**Affiliations:** 1 Cardiology Department, Getafe University Hospital, Madrid, Spain; 2 Instituto de Investigación Sanitaria del Hospital La Paz (IdiPAZ), Madrid, Spain; 3 Universidad Europea de Madrid, Madrid, Spain

**Keywords:** Conduction system pacing, Left bundle branch area pacing, Myocardial scar, Ischemic heart disease, Heart failure

**Key Teaching Points**

• The presence of a dense septal scar represents a unique adverse scenario and a major limitation for left bundle branch area pacing.
• A wide variety of delivery sheaths, high-support stylets, and short radiofrequency pulses should be considered in the preoperative planning for cases of left bundle branch area pacing in patients with septal scar.
• Mechanical septal debulking through repetitive tissue extraction using helix entanglement could be helpful as a bailout technique in cases with dense septal scar.

## Introduction

Conduction system pacing has emerged as a more physiological alternative to conventional right ventricular apical pacing and has demonstrated promising results in patients with left bundle branch block. Moreover, it is the preferred option in patients with an indication for cardiac resynchronization therapy (CRT) when coronary sinus lead implantation is not feasible or is associated with suboptimal outcomes.

However, the presence of a markedly hypertrophic or heavily scarred interventricular septum poses a significant technical challenge for achieving left bundle branch capture. We report a case of a patient with ischemic cardiomyopathy and extensive septal fibrosis in whom successful left bundle branch pacing (LBBP) was achieved through a mechanical septal debulking maneuver using the pacing lead itself.

## Case report

We describe a 66-year-old man with a history of ischemic heart disease, prior extensive anteroseptal myocardial infarction (Figure 1A), severely reduced left ventricular ejection fraction of 26%, and ventricular arrhythmias previously treated with catheter-based septal substrate ablation (Figure 1B).

**Figure 1:**
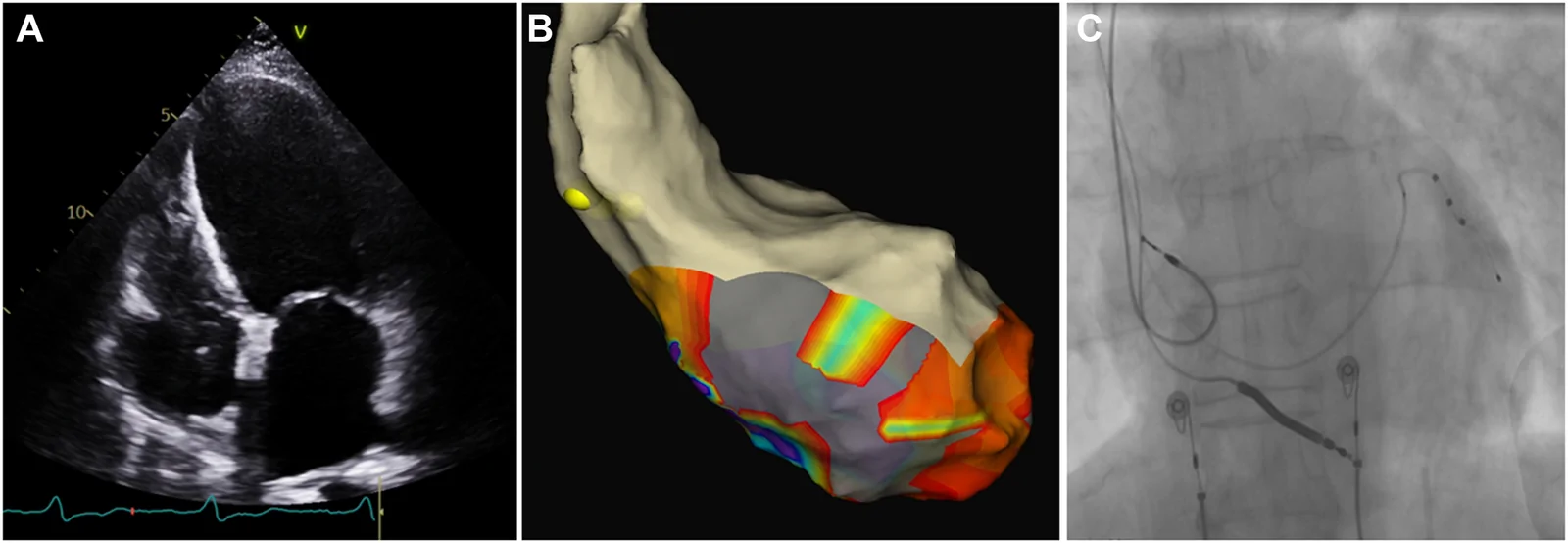
**A:** Baseline echocardiogram with old septal scarring. **B:** Voltage mapping during prior ventricular tachycardia ablation showing septal scar. **C:** Cardiac resynchronization device implanted in the patient.

Following the ablation procedure, the patient developed complete left bundle branch block. Given persistent heart failure symptoms and a left ventricular ejection fraction of <30%, an upgrade from a previously implanted single-chamber implantable cardioverter–defibrillator to a CRT system was indicated. A quadripolar left ventricular lead was implanted in a lateral branch of the coronary sinus, along with an atrial lead (Figure 1C). No additional suitable venous branches were identified in that procedure. During implantation, phrenic nerve capture was observed in the 2 distal dipoles at low output (<2.5 V) and also in the remaining proximal dipoles only at high outputs (5 and 6.5 V).

4 days after implantation, the patient reported intermittent and painful phrenic nerve stimulation, particularly in the lateral decubitus position. Device interrogation confirmed intermittent phrenic capture in the upright and left lateral positions using the distal dipoles at nominal settings. Reprogramming attempts failed to eliminate phrenic stimulation. Chest radiography showed no evidence of lead dislodgement. Therefore, extraction of the coronary sinus lead and implantation of an LBBP lead were planned.

The procedure was performed under conscious sedation. The baseline electrocardiogram at the beginning of the procedure is shown in Figure 2A. Venography demonstrated patency of the left venous system, and the left axillary vein was then cannulated. The pre-existing pocket was opened, and the coronary sinus lead was removed through simple traction without complications.

**Figure 2:**
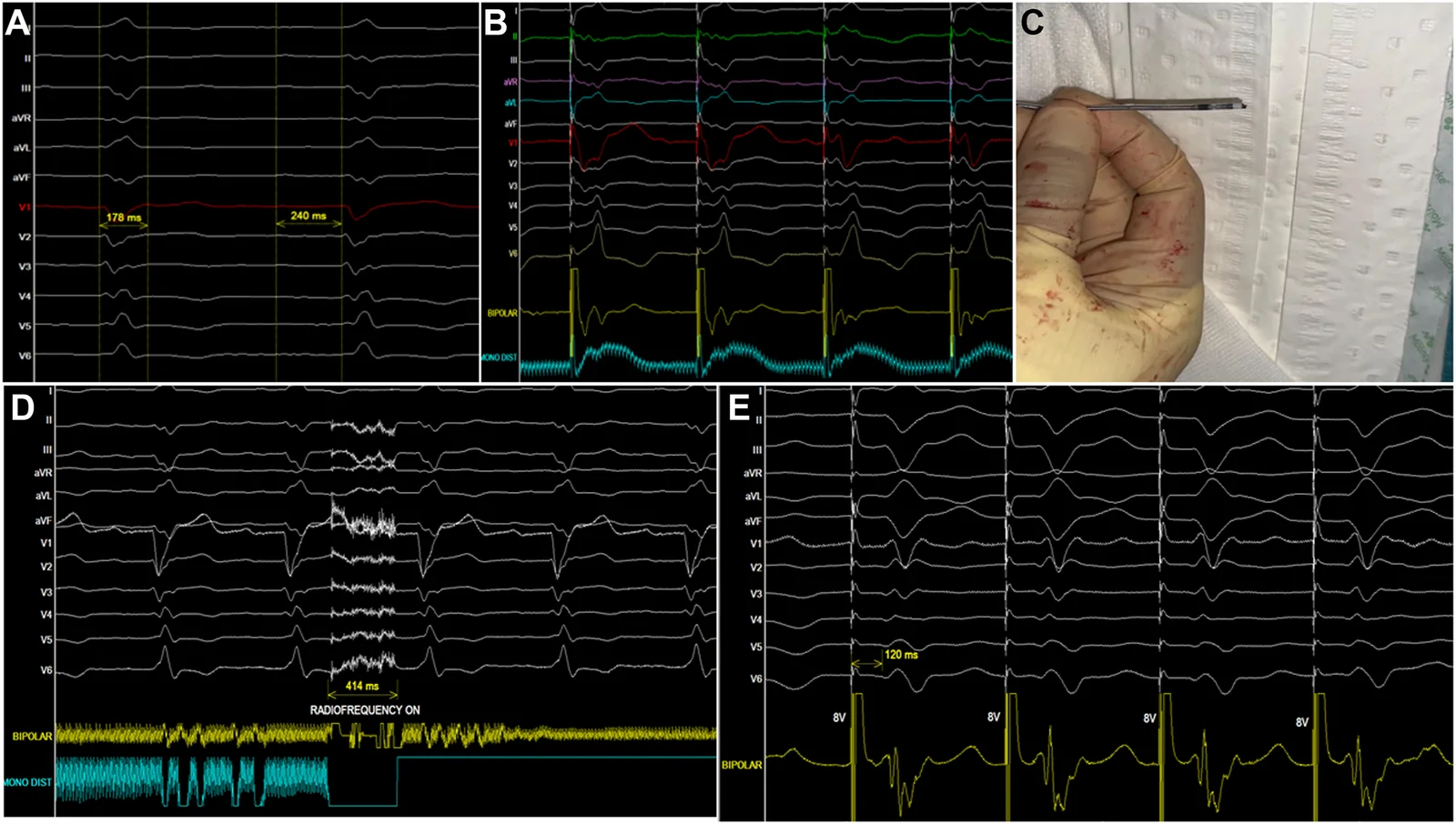
**A:** Native QRS morphology. **B:** Location with deep septal pacing. **C:** Helix damage after lead penetration attempt. **D:** Radiofrequency administration through the pacing lead. **D:** Left ventricular septal pacing with great stimulus-to-QRS delay in the scarred zone (**E**).

Using a pre-shaped delivery sheath (Selectra SelectSecure 55/42, Biotronik), a stylet-driven pacing lead (Solia S60, Biotronik) was advanced to the right ventricle. A suitable septal target site was identified by pace-mapping and fluoroscopic position, and lead screwing was initiated. The interventricular septum was noted to be extremely rigid, consistent with extensive fibrosis. A high-support stylet was exchanged for the standard stylet, allowing only limited penetration, achieving parameters consistent with deep septal pacing (Figure 2B).

During subsequent screwing attempts, significant lead entanglement within the septum was encountered, ultimately resulting in helix fracture (Figure 2C), necessitating lead replacement. Further attempts at adjacent sites with the same and other sheaths failed to achieve adequate septal penetration. As an alternative, short radiofrequency energy applications (approximately 0.5 second, 50 W) using the electrosurgical knife were delivered through the stylet at the target site (Figure 2D). Subsequent screwing attempts remained unsuccessful, resulting only in left septal pacing at high outputs with a marked stimulus-to-QRS delay (Figure 2E).

Finally, the delivery sheath was stabilized at the target region, and repeated screwing attempts were performed while applying gentle traction whenever advancement became impossible. Repetitive engagement and withdrawal of the lead without moving the sheath between attempts resulted in extraction of septal tissue fragments (Figure 3A–3B) and progressive improvement in septal penetration. Ultimately, left bundle branch stimulation, demonstrated by transition from non-selective to selective left bundle branch capture, was achieved during a final screwing attempt at the same location (Figure 3C). Electrocardiographic morphology and anatomical location were concordant with posterior fascicular pacing.

**Figure 3:**
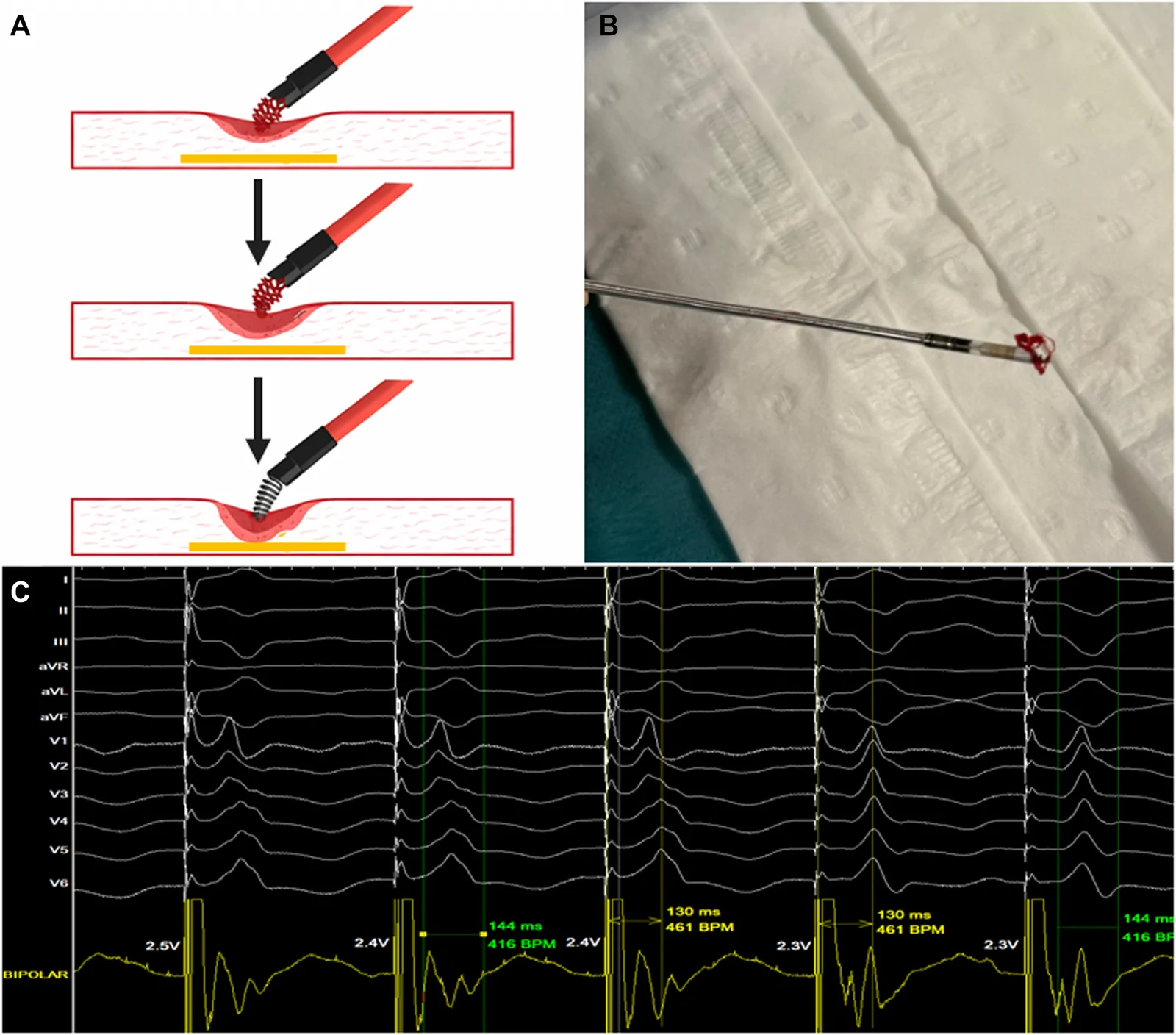
Schematic representation and actual image of the septal “debulking” technique (**A–B**) and final left bundle branch capture demonstration through transition from non-selective to selective capture of the posterior fascicle between 2.4 and 2.3 V (**C**).

The procedure was terminated without complications, and the patient was discharged on the same day with stable pacing parameters (monopolar capture threshold, 1.5 V at 0.4 ms; impedance, 678 Ω; monopolar R-wave amplitude, 5.2 mV). The final position of the lead is shown in the Supplemental Video 1. At 3-month follow-up, no device-related complications were reported. Although no echocardiographic reverse remodeling was observed, the patient remained in New York Heart Association functional class I and experienced no heart failure hospitalizations.

## Discussion

LBBP has been rapidly adopted in clinical practice and is currently used across a wide range of clinical scenarios, with high reported implantation success rates.^1^^,^^2^

Penetration of the interventricular septum remains a key part of an LBBP procedure. Identification of the optimal site to start lead screw can be achieved by different approaches, including anatomical references, fluoroscopy, pace-mapping, or endocardial mapping. During the screwing process, the lead should exhibit a smooth progression until the left subendocardial area is achieved. Some lead behavior could point to difficult scenarios in lead screwing, such as the ‘‘entanglement’’ effect—the inability to penetrate the septum because of helix entrapment on muscle fibers—or the ‘‘drill’’ effect—limited forward progression of the lead despite several successful rotations of the lead body resulting in “tunnel” creation that restricts proper lead fixation.

Dense interventricular septal fibrosis—commonly encountered in patients with cardiomyopathies or prior anteroseptal myocardial infarction—significantly limits lead penetration and has been associated with reduced LBBP implantation success.

A higher collagen content, lower myofibril density, and septal tissue heterogeneity—features commonly encountered in patients with cardiomyopathies, prior myocardial infarction, or prior ablation procedures—have been associated with failed lead advancement and lower LBBP implantation success.^3^ Several strategies have been proposed to overcome this limitation, including the development of delivery sheaths with alternative curvatures to improve septal alignment, the use of high-support stylets to enhance torque transmission, and the application of short radiofrequency pulses to facilitate septal penetration.^4^

In highly fibrotic tissue, helix entrapment can be facilitated by the limited lead advancement, predisposing to helix deformation or fracture, as observed in our case. Although reverse rotation is commonly used to resolve entrapment, tissue extraction frequently occurs upon lead withdrawal. Based on this observation, we hypothesized that intentional repetition of this maneuver at a single site could progressively remove superficial fibrotic tissue and facilitate a deeper septal penetration.

The recent LIFE-LBBAP study has reported a higher rate of lead failure among LBBP procedures with stylet-driven compared with lumenless leads.^5^ In this study, fractures in lumenless leads usually occurred proximal to the ring electrode, whereas conductor fractures in stylet-driven leads usually originated at the interelectrode spacing. In contrast with this data, we observed helix damage while using a stylet-driven lead. A high number of screw attempts could justify this observation, as in the LIFE-LBBAP study, lead fractures were observed with only 1–5 attempts.

The proposed mechanical septal “debulking’’ technique offers the advantage of being performed using standard implantation tools, without the need for additional equipment. Nevertheless, it carries a potential risk of lead damage, which may necessitate lead replacement. We encourage performing a thorough assessment, both visual and radiological, of the lead during the “debulking’’ process.

Finally, other alternatives in cases of dense septal scar and failed conventional CRT include the surgical implantation of an epicardial left ventricular lead—that can be achieved with minimally invasive surgery—or the use of new wireless endocardial devices (ie, *WISE-CRT* system).^6^

Given that this is an isolated case, no conclusions regarding efficacy or safety of this technique can be drawn from this report. However, the proposed approach may represent a feasible bailout strategy when all conventional techniques have failed.

## Conclusion

In patients with dense septal scarring requiring LBBP, mechanical septal debulking using the pacing lead itself may enable penetration of the interventricular septum and successful capture of the conduction system as a last-resort strategy.
