# Supplementary material for: Mechanical septal debulking to enable left bundle branch pacing through dense septal scar: A bailout technique
Source: HeartRhythm Case Rep. 2026 Jul 11;12(10):1251–4. doi: 10.1016/j.hrcr.2026.07.003 (PMC13687960; doi:10.1016/j.hrcr.2026.07.003)
Supplement: Supplementary file 1 [file mmc1.docx]

**SUPPLEMETARY MATERIAL:**

**Supplementary video 1. Final lead position.**
